# Twenty-four hour rhythmicity in mitochondrial network connectivity and mitochondrial respiration; a study in human skeletal muscle biopsies of young lean and older individuals with obesity

**DOI:** 10.1016/j.molmet.2023.101727

**Published:** 2023-04-14

**Authors:** Anne Gemmink, Sabine Daemen, Jakob Wefers, Jan Hansen, Dirk van Moorsel, Puji Astuti, Johanna A. Jorgensen, Esther Kornips, Gert Schaart, Joris Hoeks, Patrick Schrauwen, Matthijs K.C. Hesselink

**Affiliations:** Department of Nutrition and Movement Sciences, NUTRIM School of Nutrition and Translational Research in Metabolism, Maastricht University Medical Centre+, Maastricht, the Netherlands

**Keywords:** Day-night rhythm, Mitochondrial network integrity, Skeletal muscle, Mitochondrial function

## Abstract

**Objective:**

Mitochondrial network dynamics may play role in metabolic homeostasis. Whether mitochondrial network dynamics are involved in adaptations to day–night fluctuations in energy supply and demand is unclear. Here we visualized and quantified the mitochondrial network morphology in human skeletal muscle of young healthy lean and older individuals with obesity over the course of 24 h

**Methods:**

Muscle biopsies taken at 5 timepoints over a 24-hour period obtained from young healthy lean and older metabolically impaired obese males were analyzed for mitochondrial network integrity with confocal laser scanning microscopy. Variation of level of fragmentation over the course of the day were aligned with variation of mitochondrial respiration over the day

**Results:**

Young healthy lean individuals displayed a day–night rhythmicity in mitochondrial network morphology, which aligned with the day–night rhythmicity of mitochondrial respiratory capacity, with a more fused network coinciding with higher mitochondrial respiratory capacity. In the older individuals with obesity, the mitochondrial network was more fragmented overall compared to young healthy lean individuals and completely lacked 24 h rhythmicity, which was also true for the mitochondrial respiratory capacity

**Conclusions:**

Our data shows a paralleled rhythmicity between mitochondrial network morphology and mitochondrial oxidative capacity, which oscillates over the course of a mimicked real-life day in human skeletal muscle of young, healthy lean individuals. In older individuals with obesity, the lack of a 24-hour rhythmicity in mitochondrial network connectivity was also aligned with a lack in respiratory capacity. This suggests that 24-hour rhythmicity in mitochondrial network connectivity is a determinant of rhythmicity in mitochondrial respiratory capacity. Thus, restoring mitochondrial network integrity may promote mitochondrial respiratory capacity and hence contribute to blunting the metabolic aberrations in individuals with a disturbed 24-hour rhythmicity in metabolism, like older individuals with obesity.

## Highlights

• Myocellular mitochondrial network integrity in healthy young lean males is rhythmic.
• In young healthy males mitochondrial network and respiration align.
• Mitochondrial network integrity is not rhythmic in older metabolic impaired males.
• In older obese males mitochondrial network and respiration are not rhythmic.
• MFI provides quantitative information on mitochondrial network connectivity.

## Graphical abstract

**Figure:**
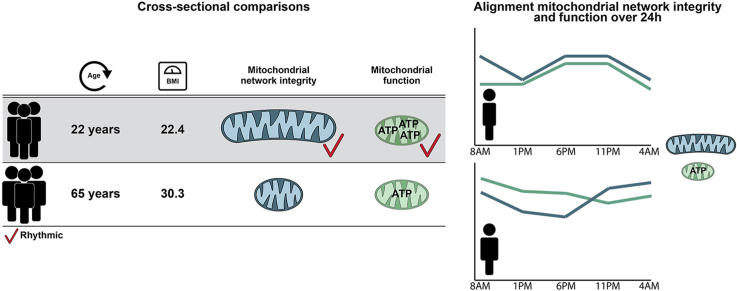

## ABBREVIATIONS

ADP: Adenosine diphosphate
BMAL1: Brain and muscle arnt-like protein 1
BMI: Body mass index
CLOCK: Circadian locomotor output cycles kaput
CRY: Cryptochrome Circadian Regulator
DRP1: Dynamin-related protein 1
FCCP: Carbonyl cyanide p-trifluoromethoxyphenylhydrazone
FIS1: Mitochondrial fission 1 protein
MEQ-SA: Moringness-eveningness questionnaire
MFI: Mitochondrial fragmentation index
MHC1: Myosin Heavy Chain Type I
MOGS: Malate, octanoylcarnitine glutamate succinate
OGIS: Oral Glucose Insulin Sensitivity
PGC-1α: Peroxisome proliferator-activated receptor gamma coactivator 1-alpha
PER2: Period Circadian Regulator 2; PINK1
PTEN: induced kinase 1
PMT: Photomultiplier tube
ROI: region of interest
TOMM20: Translocase of outer mitochondrial membrane 20

## 1 Introduction

Myocellular energy demand and supply vary throughout the day as the consequence of fluctuations in feeding and fasting, and alternating periods of exercise and rest. To deal with variations in energy demand on the long-term, (e.g. upon exercise training), mitochondrial biogenesis is stimulated via activation of the nuclear transcriptional co-activator PGC-1α [1], and thereby improving oxidative capacity. To deal with rapid fluctuations in energy demand or supply like cycles of feeding and fasting or day–night fluctuations more rapidly responsive mechanisms must be in place. In skeletal muscle, mitochondria are present in a reticular network [2] that is subject to remodeling by the processes of fusion and fission, leading to morphological and possibly functional changes of the mitochondrial network [3]. During mitochondrial fusion, the outer and inner membranes of adjacent mitochondria fuse to create elongated structures resulting in an increased connectivity of the mitochondrial network [3]. Conversely, fragmentation of the mitochondrial network via mitochondrial fission results in smaller mitochondria that are (at least partly) disconnected from the mitochondrial reticular network [3].

Originally, the process of mitochondrial fusion and fission was viewed as a mitochondrial quality control mechanism [3]. Damaged parts of the mitochondria are selectively eliminated through mitophagy, while newly formed mitochondria can fuse within the existing mitochondrial network. More recently, mitochondrial network dynamics have been suggested to play an important role in the regulation of mitochondrial respiration and metabolic homeostasis [4]. In this respect, it has also been hypothesized that mitochondrial fission is important for fueling-off excess nutrients during nutrient overload [4]. On the other hand, a highly fused mitochondrial network is associated with a high mitochondrial respiratory capacity in c. elegans [5]. In vitro, defects in mitochondrial dynamics in MEFs [6] and C2C12 cells [7] associates with reduced mitochondrial fatty acid oxidation. This strongly indicates that mitochondrial network dynamics is linked to mitochondrial respiratory capacity. In line with these findings it has been shown that in human primary myotubes of individuals with type 2 diabetes a reduced mitochondrial respiratory capacity is associated with a fragmented mitochondrial network [8]. These data are indicating that a disturbed mitochondrial network integrity may underlie mitochondrial dysfunction.

We have observed a day–night rhythmicity in mitochondrial respiratory capacity in skeletal muscle of young healthy lean individuals [9]. This rhythmicity was paralleled by oscillations in protein markers for mitochondrial fusion and fission. On the contrary, there was a lack of rhythmicity in mitochondrial respiratory capacity and markers of mitochondrial dynamics in older individuals with obesity and metabolic impairments in glucose homeostasis [10]. These data hint towards a rhythmicity of the mitochondrial reticular network, which might contribute to the rhythmicity of mitochondrial respiratory capacity in young healthy lean individuals. Here we tested this concept by quantitatively and spatially mapping skeletal muscle mitochondrial network connectivity using confocal microscopy. We studied if spatial remodeling of the mitochondrial network displays a rhythmicity that coincides with the rhythm in mitochondrial capacity. To this end, we examined biopsies obtained from individuals with confirmed rhythmicity in mitochondrial respiratory capacity (lean young healthy individuals) and biopsies from individuals not possessing rhythmicity in mitochondrial capacity (obese older individuals).

## 2 Materials and methods

### 2.1 Participants

The physiological and metabolic data of the current study were previously published by Van Moorsel et al. [9] and Wefers et al. [10]. Here, we performed additional analyses in the muscle biopsies from 12 young healthy lean Caucasian men (age ± SD: 22 ± 2 years, BMI: 22.4 ± 2.0 kg/m^2^) and 12 older metabolic impaired obese Caucasian men (age ± SD: 65 ± 9 years, BMI: 30.3 ± 2.7 kg/m^2^) that participated in those original studies. The subjects were non-smokers and engaged in ≤3 h of exercise per week. Young healthy lean participants had no active disease and were not taking any medication, while the older metabolic impaired obese participants had an impaired glucose tolerance and/or insulin sensitivity. These metabolically impaired obese participants had to fulfill at least one of the following criteria: 1) impaired fasting glucose (6.1–6.9 mmol/L); 2) impaired glucose tolerance (7.8–11.1 mmol/L 2 h after 75 g glucose intake); 3) HbA1C of 5.7–6.4%; or 4) low insulin sensitivity defined as glucose clearance rate ≤360 ml/kg/min according to the Oral Glucose Insulin Sensitivity (OGIS) model. Other inclusion criteria were regular sleep–wake cycle with sleep duration between 7 and 9 h/night, no shift work or crossing of >1 time zone within 3 months before the study. Extreme chronotypes were excluded based upon their score on a morningness-eveningness questionnaire (MEQ-SA) [11] (exclusion of scores <35 or >70). The individuals with obesity were not taking any lipid or glucose lowering drugs.

### 2.2 Study approval

The study was approved by the local Ethics Committee of the Maastricht University Medical Center and monitored by the Clinical Trial Center Maastricht. All subjects gave written informed consent before participation in the study. All procedures were performed according to the declaration of Helsinki. The original studies and measurements, excluding the histochemical analyses, were executed between November 2014 and July 2015 [9], and July 2018 and March 2019 [10] and registered at clinicaltrials.gov with identifiers NCT02261168 [9] and NCT03733743 [10].

### 2.3 Study design

Both original studies were identical in design and of a cross-sectional nature, with a one-week run in period prior to the measurements at our research facilities. The run in period consisted of a standardized lifestyle with fixed sleeping times (11 PM until 7 AM), fixed meal times (9 AM, 2 PM and 7 PM), no alcohol and no caffeinated drinks, as described before [9,10]. Adherence to this standardized lifestyle prior to the measurements was checked using actigraphy and a sleep diary. Meal times and composition were registered in a food diary for 1 week for the three days prior to visiting the lab. During the study, subjects remained under strictly standardized conditions in the laboratory for a total of 44 h (from noon on day 1–8 AM on day 3). Meals and activities were scheduled in order to mimic a real-life situation. Fixed meal times were the same as during the run in period, 9 AM, 2 PM and 7 PM. Physical activity was prescribed 1 h after each meal, consisting of a 15 min light intensity walk and 15 min of standing. During their whole stay, participants resided in a metabolic chamber. A detailed description can be found in the original study publications [9,10]. Muscle biopsies were obtained at 8 AM, 1 PM, 6 PM, 11 PM and 4 AM. To exclude acute effects of a meal intake or physical activity bout, muscle biopsies were taken before breakfast, lunch and dinner.

### 2.4 Study meals

No drinks other than water were allowed between the meals. Participants were provided with meals according to their caloric requirements for the last 2 days of the run-in period [9,10]. Caloric intake during the study period was calculated by multiplying the sleeping metabolic rate of the first study night with an activity factor of 1.5. The daily macronutrients consisted of ∼52 energy% carbohydrates, ∼31 energy% fat (∼9% saturated), and ∼14 energy% as protein. Breakfast accounted for ∼21 energy%, lunch for ∼30 energy% and dinner for ∼49 energy%.

### 2.5 Skeletal muscle biopsies and respirometry

Muscle biopsies were obtained from the m. vastus lateralis by using the Bergström method [12]. Local anaesthetization was done with 1% lidocaine, without epinephrine. Incisions for the five biopsies were at least 2 cm apart, moving from distal to proximal. Biopsies were taken alternately between both legs, with the first leg randomized. Part of the muscle biopsy was placed on a drop of TissueTek on an object glass slide and immediately frozen in melting isopentane and stored in −80 °C until further analysis. Another part of the same biopsy was freshly used for the preparation of permeabilized muscle fibers and subsequent measurement of mitochondrial respiratory capacity upon several substrates (malate, octanoylcarnitine, ADP, glutamate, succinate, and carbonylcyanide p-trifluoromethoxyphenylhydrazone (FCCP)) using an Oxygraph (OROBOROS Instruments), as described before [9].

### 2.6 Histochemical analyses

To evaluate mitochondrial network morphology, five μm thick sections were cut and mounted on glass slides. In the cryostat, muscle biopsies were mounted in such a way that longitudinal sections could be cut. This was checked for every section with a light microscope available next to the cryostat. To minimize variability in staining, all five time points of the same participant were mounted on the same glass slide. Sections were fixed by incubating for 30 min in 3.7% formaldehyde (104003, Merck, Darmstadt, Germany), followed by permeabilization using 0.5% Triton X100 (648466, Merck, Darmstadt, Germany). Subsequently, sections were incubated for 60 min with primary antibodies against caveolin as a marker protein of the plasma membrane to detect individual muscle fibers (610421, BD Biosciences, Franklin Lakes, New Jersey, USA), translocase of the outer mitochondrial membrane 20 (TOMM20) as a mitochondrial marker protein (Ab186734, Abcam, Cambridge, UK) and myosin heavy chain type I (MHC1) to detect muscle fiber typology (A4.840, Developmental Studies Hybridoma Bank, Iowa City, Iowa, USA). Thereafter, sections were incubated for 2 h with appropriate secondary antibodies labeled with AlexaFluor 405, 488 and 555 (Invitrogen-ThermoFisher, Groningen, The Netherlands). Finally, sections were mounted with Mowiol (475904, Merck, Darmstadt, Germany) and covered with #1 coverslips.

### 2.7 Image acquisition and analysis

Images were obtained on a Leica TCS SP8 microscope in confocal mode with a 100× oil immersion 1.4 N.A. objective in a blinded fashion. All longitudinal fibers present in a sample were imaged once. This resulted in an average of 11.5 ± 0.4 fibers imaged per sample in the young healthy lean group, and 16.4 ± 0.5 fibers imaged per sample in the older metabolic impaired obese group. On each image there is/are typically 1 to 2 longitudinal fiber(s) present. Images were taken with 2048 × 2048 pixels format resulting in a pixel size of 57 by 57 nm. TOMM20-AF405 was imaged using a 405 laser line, while caveolin-AF488 and MHC1-AF555 were imaged using a white light laser (470–670) at a wavelength of 488 and 555, respectively. Emission was detected at 425–460 nm, 500–530 nm and 565–630 nm for TOMM20-AF405, caveolin-AF488 and MHC1-AF555, respectively, using a PMT detector. Subsequently, images were deconvolved using Huygens Professional Software (Scientific Volume Imaging B.V., Hilversum, The Netherlands) in the batch processing modus. Image analysis was performed using ImageJ (NIH, Bethesda, Maryland, USA) using in house written scripts. Mitochondrial images were background corrected and filtered using Gaussian and median filtering. Binary images were created by automatic thresholding using the ‘make binary’ command in ImageJ for both mitochondrial networks and cell membranes. Subsequently, longitudinal sectioned muscle fibers were selected as regions of interest (ROIs) based on the binary images of the cell membranes and stored in the ROI manager. For each ROI a particle analysis was performed using the ‘analyze particles’ command in ImageJ to determine total mitochondrial area and number of mitochondrial network particles per fiber. Type I and II muscle fibers were distinguished using the MHC1 staining. Based on Halling et al. [13], the mitochondrial fragmentation index (MFI) was calculated for each fiber by dividing the total mitochondrial network particle count by the total mitochondrial area. MFI for all fibers together was calculated as a weighted average of type I and type II MFI values, taking into account fiber type ratio of all 5 muscle biopsies taken per individual, which was determined using a Nikon E800 fluorescence microscope on cross sections prior to confocal imaging and based on the MHC1-AF555 staining. To assess whether oscillations in MFI were aligned with mitochondrial respiration, for visualization purposes the inverse of MFI was plotted together with mitochondrial respiration. Both parameters were normalized to the average of all 5 timepoints. Since the MFI is calculated as the mitochondrial network particle count divided by total mitochondrial area, the inverse is total mitochondrial area divided by mitochondrial count, which equals mitochondrial network particle size.

Mitochondrial network integrity/fragmentation status can be described in addition to the MFI by shape descriptors such as circularity, geodesic diameter and geodesic elongation. For validation of the MFI as a parameter for mitochondrial network integrity/fragmentation status, we compared the MFI with these shape descriptors. In Supplemental Figure 1 the different shape descriptors used are visualized and how to interpret their values. Circularity of the mitochondrial network particles was determined in a subset of the participants (n = 12). A characteristic of a fragmented mitochondrial network is the punctate and round shaped mitochondria, while mitochondria of a fused mitochondrial network are elongated shaped. Circularity values heading towards 1 are round shape network particles and indicate thus a more fragmented network, while values closer to 0 are elongated particles and indicative of a more fused mitochondrial network. In addition, in the same subset of the participants (n = 12) we acquired higher magnification confocal images (100× oil immersion 1.4 N.A. objective, 5× optical zoom, 23 nm × 23 nm pixel size). These images were quantified with ImageJ using the MorphoLibJ plugin [14] for other shape descriptors, such as the geodesic diameter and the geodesic elongation. The geodesic diameter is the largest geodesic distance between two points within a structure. The geodesic elongation is the ratio of the geodesic diameter over the diameter of the largest inscribed circle within a structure. A ratio of 1 indicates a perfect circle, and thus a fragmented mitochondrial network. The longer the geodesic elongation, the more elongated the structures are and thus a more fused mitochondrial network. In addition, mitochondrial networks were visually scored by two independent and blinded observers in 160 images obtained in a pilot study.

### 2.8 Statistics

Data are presented as mean ± SEM (standard error of the mean). Statistical analyses were performed with the use of IBM Statistical Package for Social Sciences, version 28 (IBM Corp., Armonk, NY, USA). The effect of time on outcome variables was analyzed with linear mixed models. In case of significant effect of time for the MFI, we tested for rhythmicity using the JTK_CYCLE package in R 3.6.3 for MAC [15] as a post-hoc analysis and reported the Bonferroni adjusted p-value. To test for statistical significant differences between groups an independent t-test was performed. A two-way repeated measures ANOVA was performed to analyze for statistical differences for MFI between fiber types with group as a between subject factor and fiber type as within subject factor. When a significant interaction effect was observed a Bonferroni post-hoc test was performed. For validation of the MFI against other parameters describing mitochondrial network integrity we performed simple linear regression. To examine inter-observer reliability, which was used to examine if MFI derived scores correlate with scores obtained after visual inspection by experienced histologists, the cores after visual inspection by the two independent and blinded observers was analyzed using the Cronbach's alpha. Statistical significance was defined as a p-value <0.05.

## 3 Results

### 3.1 Validation of the mitochondrial fragmentation index as a measure for mitochondrial network integrity

Since the MFI was the main outcome parameter for our study, we first validated the MFI against visually obtained scores by two independent well trained observers (Supplemental Figure 2A and Supplemental Figure 3). This resulted in a Cronbach's alpha of 0.794 which is considered as a good agreement between observers (n = 160 images, pilot study). In addition, we used a different semi-automated approach that gives mitochondrial shape descriptors, (circularity geodesic diameter, and geodesic elongation, Supplemental Figures 2B–D) in 12 participants from this study. For these we also analyzed if MFI correlates with these shape descriptors, which it did (Supplemental Figure 2B, C and D). Both geodesic parameters were sensitive enough to pick up clear differences in the mitochondrial network integrity (Supplemental Figure 2E). Thus, all these shape descriptors correlated with the 10.13039/100014329MFI, and support the notion that 10.13039/100014329MFI is a proper measure for mitochondrial network integrity (Supplemental Figures. 2A–2D).

### 3.2 Participant characteristics

Characteristics of the participants have been published before [9,10] and can be found in the Supplemental Table 1.

### 3.3 Mitochondrial network integrity has a 24-hour rhythm in young healthy lean individuals

**Figure 1:**
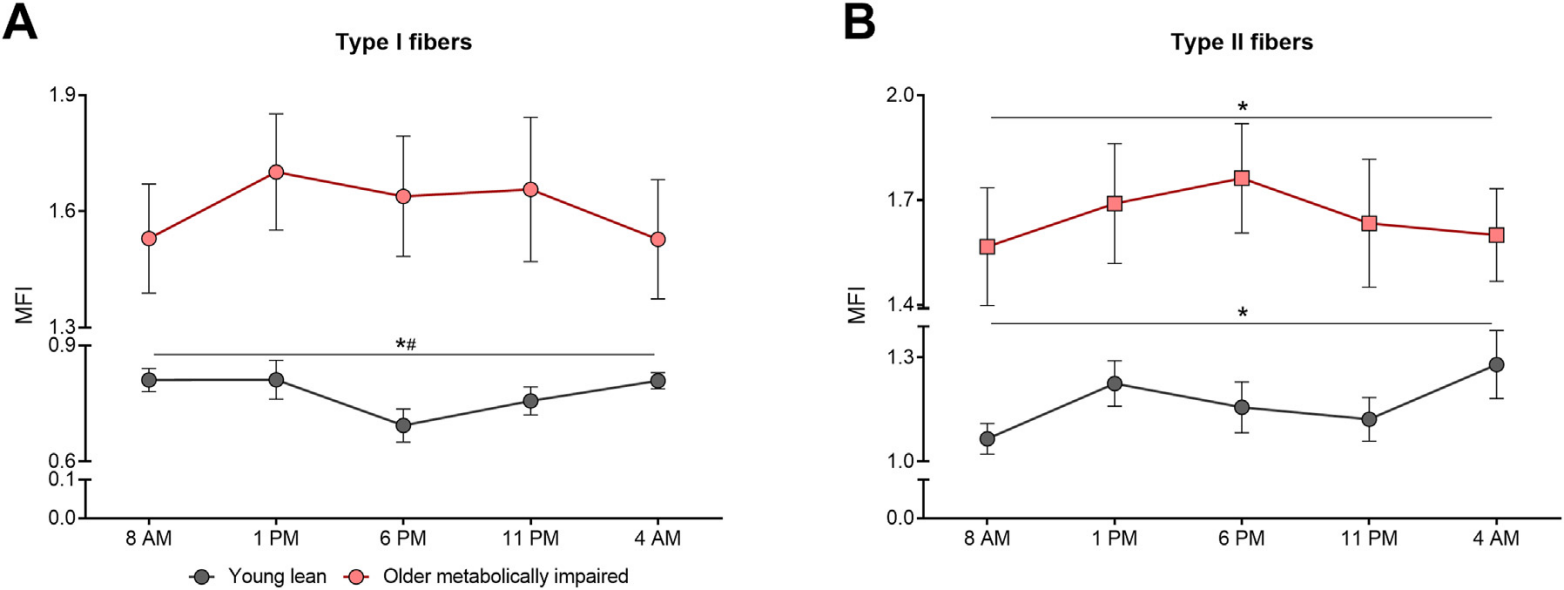
**Mitochondrial network integrity has a 24-hour rhythm in young healthy lean individuals.** Mitochondrial fragmentation index (MFI) at five successive time points over a 24-hour period in type I (A) and type II (B) muscle fibers. Data are presented as mean ± SEM. ∗ indicates a significant time effect obtained from the linear mixed models analysis; # indicates a significant rhythmicity obtained from the JTK-cycle analysis p < 0.05.

**Figure 2:**
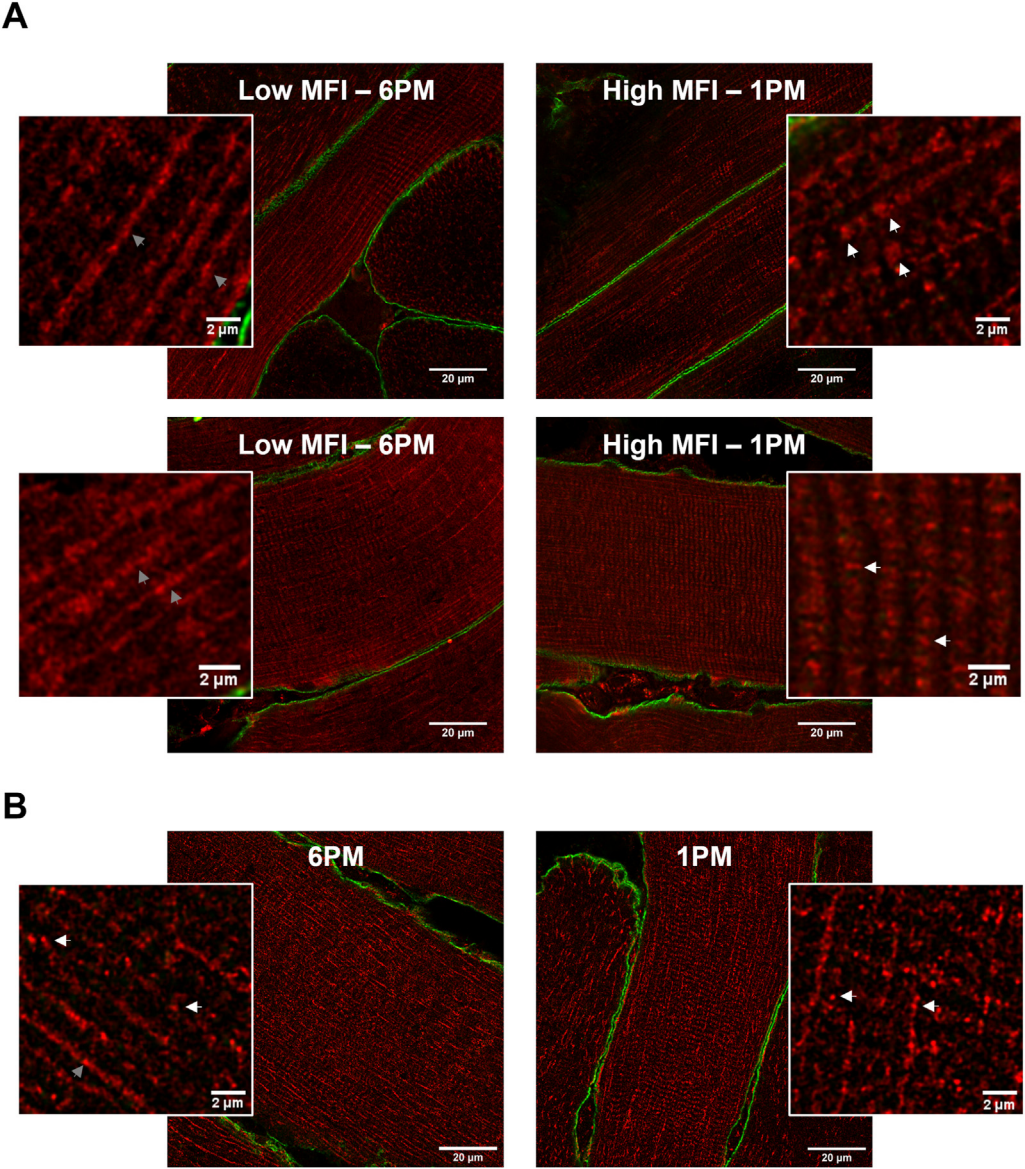
**Visualization of day–night differences in mitochondrial network morphology in young healthy lean individuals.** Representative images and corresponding zoomed images of the mitochondrial network in type I fibers in longitudinal view at the time points with the highest MFI (right) and lowest MFI (left) of two young healthy lean participants (A) and of the same timepoints of an older participant with obesity and metabolic impairments (B). Mitochondria are visualized in red and cellular membrane in green. Arrows indicate either elongated mitochondrial structures (low MFI, left, grey arrows) or punctate mitochondrial structures (high MFI, right, white arrows). At the time points with a lower MFI, representing less fragmentation, more elongated mitochondrial structures could be observed in the young healthy lean individuals. On the contrary, the time points with highest MFI in the young healthy lean individuals displayed a more punctate mitochondrial structures, reflective of fragmentation of the mitochondrial network. The mitochondrial network in the older individuals with obesity and metabolic impairments showed at both timepoints a clearly more punctate mitochondrial structures compared to the young healthy lean individuals.

When analyzing the MFI for each fiber type separately we observed a significant time effect in MFI in type I and type II fibers in the young healthy lean individuals (type I fibers: p < 0.05; type II fibers: p < 0.01, Figure 1, Figure 2). JTK_CYCLE analysis confirmed a significant 24-hour rhythmicity in the MFI in oxidative type I fibers (p < 0.01), but not in glycolytic type II fibers (p = 1.00). The lowest mitochondrial fragmentation was observed at 6 PM in type I fibers (Figure 1, Figure 2A). In the older individuals with obesity and metabolic impairments we observed a significant time effect only in type II fibers (p < 0.01, Figure 1B), without significant 24-hour rhythmicity (p = 1.00, JTK_CYCLE). These data indicate that skeletal muscle of young, healthy lean individuals display 24-hour rhythmicity in mitochondrial network integrity in oxidative type I fibers, and that such rhythmicity is absent in older, older individuals with obesity and metabolic impairments and in type II fibers in both groups.

### 3.4 Mitochondrial network integrity aligns the 24-hour rhythm in mitochondrial respiratory capacity in young healthy lean individuals

**Figure 3:**
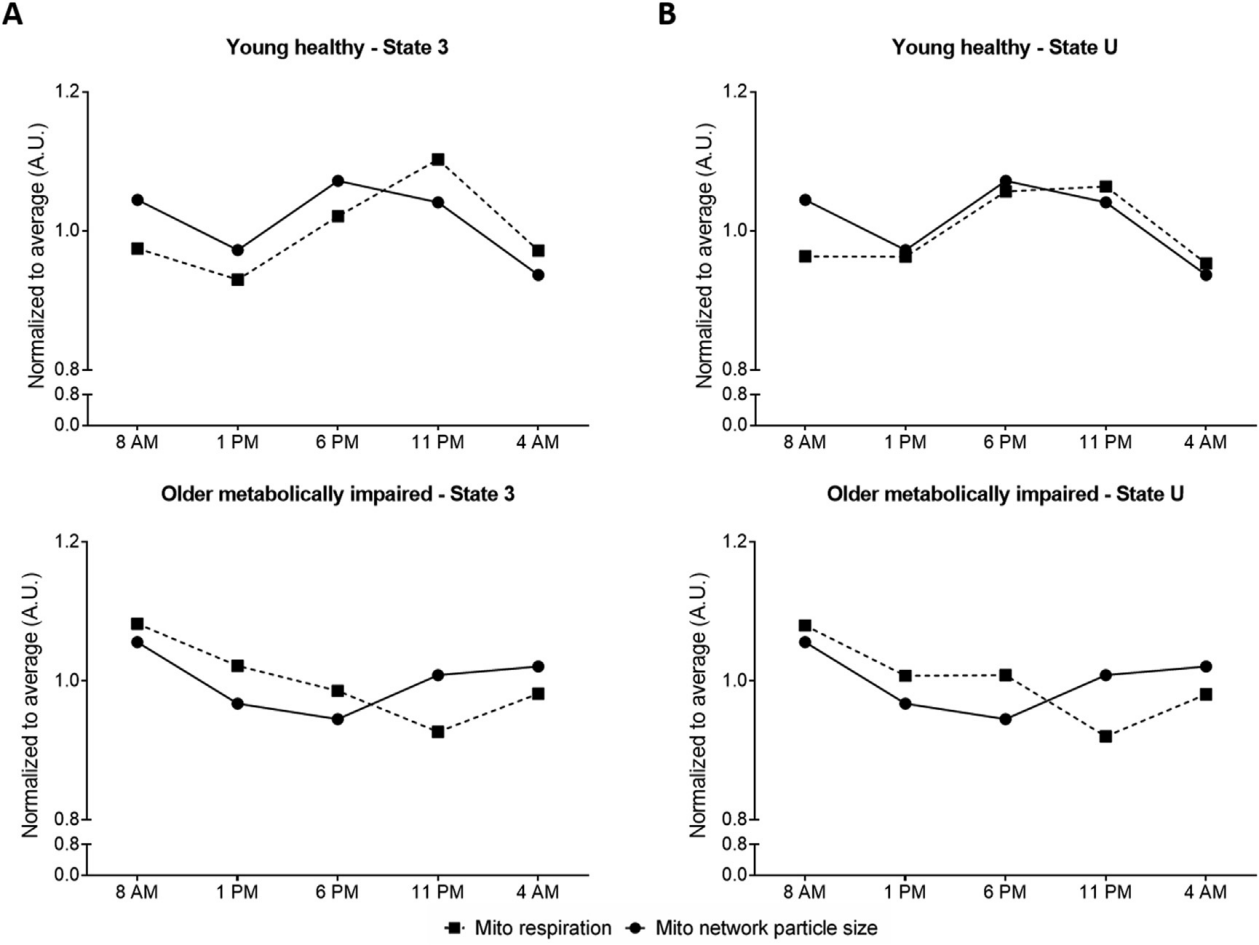
**Mitochondrial network integrity aligns the 24-hour rhythm in mitochondrial respiratory capacity in young healthy lean individuals.** (A) Alignment of plots of the inverse of the MFI, i.e. mitochondrial network particle size, and state 3 MOGS respiration for young healthy lean individuals (upper panel) and older individuals with obesity and metabolic impairments (lower panel). (B) Alignment of plots of the inverse of the MFI, i.e. mitochondrial network particle size, and state uncoupled respiration for young healthy lean individuals (upper panel) and older individuals with obesity and metabolic impairments (lower panel). For visualization reasons the inverse of the MFI is chosen to plot in the same graph as the mitochondrial respiration states. Data in all panels are normalized to the average of all five time points.

We previously demonstrated a day–night rhythm in muscle mitochondrial oxidative capacity, as assessed by high-resolution respirometry in permeabilized muscle fibers from the same biopsies as in the current study, in young healthy lean individuals [9], but not in older metabolically impaired obese participants [10]. To assess if oscillations in mitochondrial network integrity underlie the day–night rhythm in mitochondrial respiratory capacity, we plotted the average mitochondrial network particle size of the type I and II fibers combined, which equals the inverse of the MFI, together with the previously obtained data on mitochondrial respiratory capacity (Figure 3). Since the mitochondrial respiratory capacity was measured in a mixture of type I and II fibers, we used the average mitochondrial network particle size of type I and II fibers combined for these analyses. Muscle fiber type distribution was similar between groups (46 ± 3% vs. 43 ± 3% type I fibers for respectively young healthy lean vs. older metabolically impaired obese, p = 0.41). We hypothesized that a more fused mitochondrial network would coincide with a higher mitochondrial respiratory capacity. Interestingly, in young healthy lean individuals we observed that mitochondrial network integrity followed a similar rhythmic pattern as mitochondrial ADP-stimulated respiration (state 3 MOGS, Figure 3A). Even more striking, the day–night rhythm in mitochondrial network integrity presented an almost perfect overlay with the 24-hour rhythm in maximally uncoupled state U respiration in young healthy lean individuals (Figure 3B). In the older individuals with obesity and metabolic impairments in which we did not observe 24-hour rhythmicity, neither in mitochondrial network integrity nor mitochondrial function, the overlay was less obvious. Therefore, these data show that a fused and intact mitochondrial network rather than a fragmented network is compatible with a higher mitochondrial oxidative capacity in human skeletal muscle. Furthermore, these data indicate that 24-hour rhythmicity in mitochondrial respiratory capacity may be due to the rhythmicity in mitochondrial network integrity in young healthy lean skeletal muscle.

### 3.5 Mitochondrial networks are more fragmented in the older individuals with obesity and metabolic impairments

**Figure 4:**
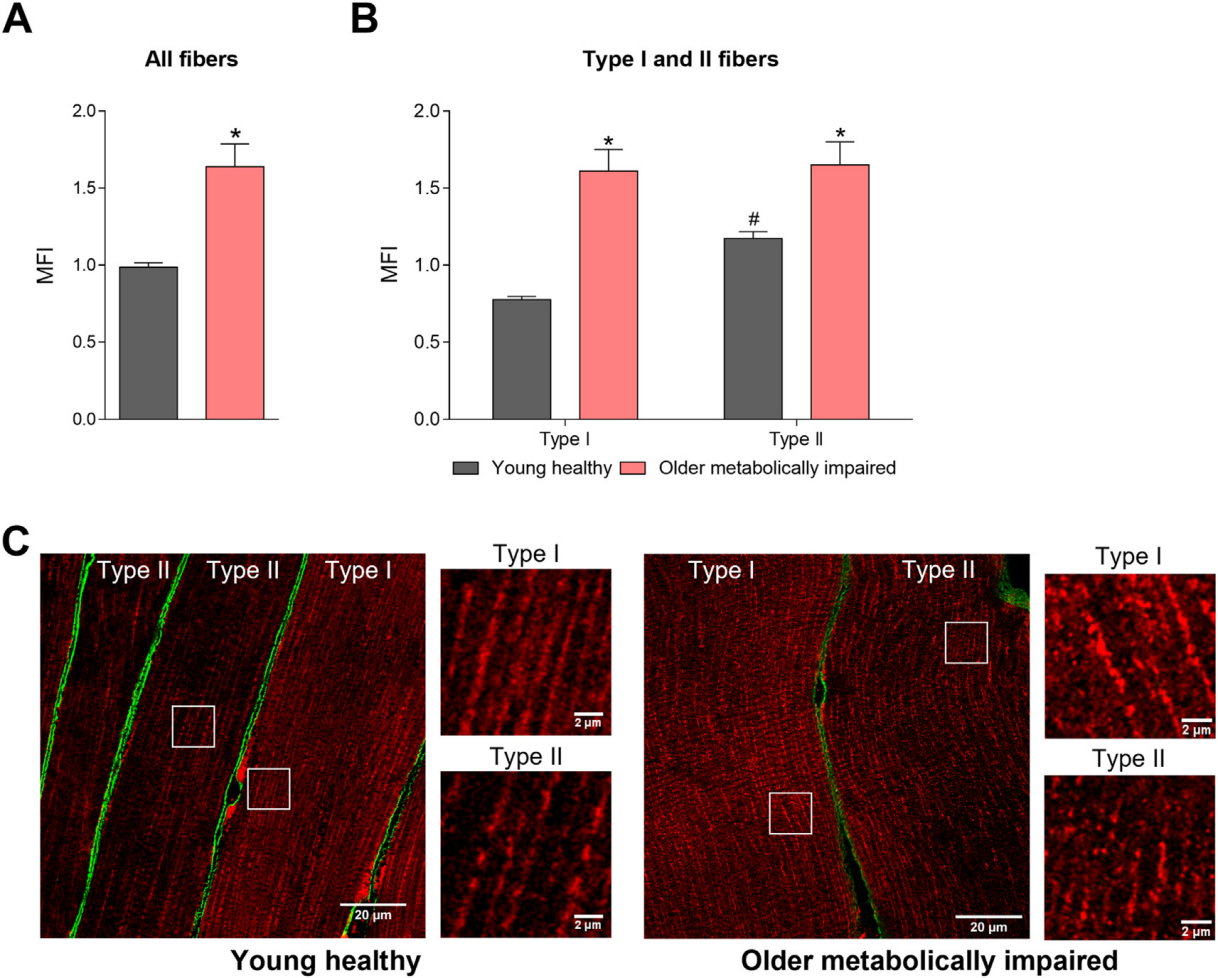
**Mitochondrial networks are more fragmented in the older individuals with obesity and metabolic impairments.** Average mitochondrial fragmentation index (MFI) of the five successive timepoints over a 24-hour period for all muscle fibers together (A) and type I and II muscle fibers separately (B). (C) Representative images of type I and II fibers showing a more fragmented mitochondrial network in type II fibers of young healthy lean (left panels) and older individuals with obesity and metabolic impairments (right panels). Mitochondria are visualized in red and cellular membrane in green. Data are presented as mean ± SEM. ∗ indicates differences between groups with p < 0.05 and #p < 0.05 for difference with type I fibers.

We have previously shown that mitochondrial function is lower in pre-diabetes and type 2 diabetes volunteers [16], and that mitochondrial respiratory capacity reduces with ageing [17]. Also in the current study, mitochondrial function was lower in the older individuals with obesity and metabolic impairments compared to the young, healthy lean individuals. In line with the lower mitochondrial oxidative capacity in this population, we here show that the MFI in the older individuals with obesity and metabolic impairments is higher compared to the young healthy lean participants over the whole day, indicating a more fragmented mitochondrial network (Figure 1). When averaged over all five timepoints, the MFI was significantly higher in older individuals (all fibers: 0.99 ± 0.03 vs. 1.64 ± 0.15, p < 0.001; type I: 0.78 ± 0.02 vs. 1.61 ± 0.14, p < 0.001; type II: 1.17 ± 0.04 vs. 1.65 ± 0.15, p < 0.01 in young healthy lean vs. older individuals with obesity and metabolic impairments, Figure 4). These data support the idea that a fragmented mitochondrial network morphology may underly the reduced mitochondrial function as observed in the insulin resistant state.

### 3.6 Mitochondrial network integrity differs between type I and type II fibers in young healthy lean individuals

Since we analyzed the fragmentation index in type I and type II fibers separately in the current study, our data set also allows to examine putative differences in mitochondrial network integrity between muscle fiber types. A significant interaction effect was observed for fiber type and group (p < 0.01). Post hoc analyses showed that the MFI, averaged over the whole day, was higher in type II fibers compared to type I fibers in the young healthy lean individuals (0.78 ± 0.02 vs. 1.17 ± 0.04, Type I vs. Type II, p < 0.01, Figure 4B,C). Interestingly, in the older individuals with obesity and metabolic impairments this difference in mitochondrial network integrity between fiber types was not observed (1.61 ± 0.14 vs. 1.65 ± 0.15, Type I vs. Type II, p = 0.245, Figure 4B,C), probably due to the overall high level of mitochondrial fragmentation in these individuals (Figure 4B). These data suggest that mitochondrial network integrity is not only related to metabolic phenotype, but also to muscle fiber type in healthy lean muscle.

## 4 Discussion

Here we aimed to examine whether the previously observed day–night rhythmicity in skeletal muscle mitochondrial respiratory capacity of young, lean subjects was paralleled by rhythmicity in mitochondrial network integrity. We observed that mitochondrial network integrity in type I muscle fibers displays a 24-hour rhythm and that the variation in mitochondrial network integrity over the day followed almost the exact same pattern of maximal mitochondrial respiratory capacity (state U) in young healthy lean individuals. Interestingly, in line with the previously observed lack of 24-hour rhythmicity in mitochondrial capacity, no rhythmicity in network integrity was observed in older, individuals with obesity and metabolic impairments. These data may suggest that mitochondrial network integrity plays an important role in regulating oscillations in mitochondrial function in humans under healthy and metabolically disturbed conditions such as insulin resistance

Previously, we observed in young healthy lean individuals [9] that proteins involved in regulating mitochondrial dynamics (FIS1 and PINK1) varied over the day and followed a similar pattern as observed for mitochondrial respiratory capacity. In the older individuals with obesity and metabolic impairments these proteins did not vary over the day and did not follow a similar pattern in mitochondrial respiratory capacity [10]. Here we showed that day–night rhythmicity in mitochondrial network integrity aligns with the rhythm observed in mitochondrial respiratory capacity in skeletal muscle of young healthy lean individuals. These data support the notion that a more fused mitochondrial network associates with a higher mitochondrial oxidative capacity in young healthy lean human skeletal muscle. The rhythmicity in mitochondrial network integrity and alignment with mitochondrial respiratory capacity was not observed in the older individuals with obesity and metabolic impairments. This lack of rhythmicity – and overall lower levels of mitochondrial network integrity in the older individuals with obesity and metabolic impairments as compared to young, healthy lean individuals – suggest that mitochondrial network dynamics are a target to improve mitochondrial function and alleviate metabolic aberrations such as insulin resistance. In that context, it has been shown previously that 12-weeks of exercise training, which improves insulin sensitivity and mitochondrial function, also remodeled the balance of fusion and fission protein expression towards mitochondrial fusion [18]. In addition, exercise training lowers DRP1 fission activity [19]. Furthermore, 4 months of exercise training enhanced the protein balance towards a mitochondrial fusion phenotype and larger mitochondria [20]. In addition, a more fused mitochondrial network is observed in endurance trained athletes compared to individuals with type 2 diabetes [21]. Moreover, a protein expression/phosphorylation pattern supportive for mitochondrial fusion is observed in endurance trained individuals compared to age matched overweight individuals [20]. This indicates that exercise training (interventions) induce(s) a more fused mitochondrial network.

Besides mitochondrial network integrity being rhythmic in young healthy lean, but not in older individuals with obesity and metabolic impairments, we also observed in the current study that the mitochondrial network showed a higher level of fragmentation in the older individuals with obesity and metabolic impairments irrespective of the time of day. This is in line with previous findings showing impaired mitochondrial network connectivity in skeletal muscle of type 2 diabetes patients [[21], [22], [23]]. This is consistent with lower mitochondrial respiratory capacity in these volunteers [16]. In addition, an increased mitochondrial fragmentation upon lipid infusion-induced insulin resistance has been reported recently [24]. These data therefore strongly supports the idea that mitochondrial network morphology is linked to the metabolic phenotype and age of the individual.

Our analysis also allowed us to examine differences in mitochondrial network integrity between type I and type II muscle fibers. In young healthy lean individuals, we observed a more fragmented mitochondrial network in type II versus type I fibers. This matches previous observations in mouse skeletal muscle showing a more elongated network in oxidative type I fibers and a more punctate mitochondrial network in glycolytic type II fibers [25]. In human muscle fibers, it has previously been observed that both type I and type II muscle fibers display a tubular interconnected network of mitochondria, with mitochondrial tubes appearing wider in type I muscle fibers [22], indicating that differences in mitochondrial networks between fiber types also exist in humans. Together, these data suggest that mitochondrial fragmentation is not only related to metabolic phenotype and age, but also to muscle fiber type.

Although it is tempting to speculate on the origin of the apparent differences in mitochondrial network connectivity and capacity between the young lean and the older obese group, one should not that this study was not designed to do so. Most likely body composition, ageing and non-specified impairments in metabolic health contribute to compromised mitochondrial network fragmentation and lack of rhythmicity, but we have no data to support this notion. Indirect support from human primary myotubes obtained from age-matched healthy lean and severely individuals with obesity showed that an obese or type 2 diabetic phenotype of the donor comes with a more fragmented mitochondrial network compared to the network in age-matched healthy lean individuals [8]. In addition, in c. elegans it has been show that ageing is associated with an increase in mitochondrial network fragmentation, a process can be slowed down with exercise training [26]. This indicates that maintaining dynamics in mitochondrial network connectivity promotes healthy ageing. Notably, in the present study only males have been investigated. Given the observations that maximal fat oxidation in males possesses diurnal variation [27] while no such variation has been observed in women [28], extrapolation of the current data to females should be done with care.

In summary, this study shows that mitochondrial network morphology may underlie the day–night rhythm previously observed in muscle mitochondrial respiration capacity in lean young individuals. The lack of rhythmicity in mitochondrial respiratory capacity along with the absence of rhythmicity in mitochondrial network connectivity in obese older individuals supports this notion. The origin of the differences in rhythmicity between the young lean individuals and the older individuals with obesity, remains to be elucidated. Restoring mitochondrial network integrity and promoting connectivity may alleviate metabolic aberrations in individuals with a disturbed 24-hour rhythmicity in metabolism such as observed with ageing and type 2 diabetes.

## Author contributions

AG, SD, PA, EK and GS performed experiments and analysis. Jha, DvM and JW performed the underlying human studies. SD, DvM, PS and MKCH designed the current analysis. JHa, JJ and JHo performed the respiration measurements and analysis. AG, SD, JW, PS, MKCH interpreted the data and wrote the manuscript.

## Funding

This work is partly financed by the Netherlands Organization for Scientific Research ((TOP 40-00812-98-14047 to PS). SD is supported by 10.13039/501100003092Dutch Diabetes Research Foundation (grant DF 2014.00.1756). SD and JHa were supported by the NUTRIM—School of Nutrition and Translational Research in Metabolism– 10.13039/501100003246NWO Graduate Program financially supported from Netherlands Organization for Scientific Research (022.003.011). JH was supported by a Vidi grant (917.14.358) for innovative research from the Netherlands Organization for Scientific Research (10.13039/501100003246NWO). The work of AG, SD, JW, PS and MKCH is partly supported by the Netherlands Cardiovascular Research Initiative: an initiative with support of the Dutch 10.13039/100002129Heart Foundation (CVON2014-02 ENERGISE).

## Data sharing

Data underlying the findings described in this manuscript may be available upon request.

## Conflict of interest

The authors have no conflict of interest to disclose.

## Data Availability

Data underlying the findings described in this manuscript may be available upon request.

## References

[1] Wu Z., Puigserver P., Andersson U., Zhang C., Adelmant G., Mootha V. (1999). Mechanisms controlling mitochondrial biogenesis and respiration through the thermogenic coactivator PGC-1. Cell.

[2] Shaw C.S., Jones D.A., Wagenmakers A.J. (2008). Network distribution of mitochondria and lipid droplets in human muscle fibres. Histochem Cell Biol.

[3] Dahlmans D., Houzelle A., Schrauwen P., Hoeks J. (2016). Mitochondrial dynamics, quality control and miRNA regulation in skeletal muscle: implications for obesity and related metabolic disease. Clin Sci.

[4] Liesa M., Shirihai O.S. (2013). Mitochondrial dynamics in the regulation of nutrient utilization and energy expenditure. Cell Metabol.

[5] Hartman J.H., Smith L.L., Gordon K.L., Laranjeiro R., Driscoll M., Sherwood D.R. (2018). Swimming exercise and transient food deprivation in Caenorhabditis elegans promote mitochondrial maintenance and protect against chemical-induced mitotoxicity. Sci Rep.

[6] Rambold A.S., Cohen S., Lippincott-Schwartz J. (2015). Fatty acid trafficking in starved cells: regulation by lipid droplet lipolysis, autophagy, and mitochondrial fusion dynamics. Dev Cell.

[7] King W.T., Axelrod C.L., Zunica E.R.M., Noland R.C., Davuluri G., Fujioka H. (2021). Dynamin-related protein 1 regulates substrate oxidation in skeletal muscle by stabilizing cellular and mitochondrial calcium dynamics. J Biol Chem.

[8] Gundersen A.E., Kugler B.A., McDonald P.M., Veraksa A., Houmard J.A., Zou K. (2020). Altered mitochondrial network morphology and regulatory proteins in mitochondrial quality control in myotubes from severely obese humans with or without type 2 diabetes. Appl Physiol Nutr Metabol.

[9] van Moorsel D., Hansen J., Havekes B., Scheer F., Jorgensen J.A., Hoeks J. (2016). Demonstration of a day-night rhythm in human skeletal muscle oxidative capacity. Mol Metabol.

[10] Wefers J., Connell N.J., Fealy C.E., Andriessen C., de Wit V., van Moorsel D. (2020). Day-night rhythm of skeletal muscle metabolism is disturbed in older, metabolically compromised individuals. Mol Metabol.

[11] Horne J.A., Ostberg O. (1976). A self-assessment questionnaire to determine morningness-eveningness in human circadian rhythms. Int J Chronobiol.

[12] Bergstrom J. (1975). Percutaneous needle biopsy of skeletal muscle in physiological and clinical research. Scand J Clin Lab Invest.

[13] Halling J.F., Ringholm S., Olesen J., Prats C., Pilegaard H. (2017). Exercise training protects against aging-induced mitochondrial fragmentation in mouse skeletal muscle in a PGC-1alpha dependent manner. Exp Gerontol.

[14] Legland D., Arganda-Carreras I., Andrey P. (2016). MorphoLibJ: integrated library and plugins for mathematical morphology with ImageJ. Bioinformatics.

[15] Hughes M.E., Hogenesch J.B., Kornacker K. (2010). JTK_CYCLE: an efficient nonparametric algorithm for detecting rhythmic components in genome-scale data sets. J Biol Rhythm.

[16] Phielix E., Meex R., Moonen-Kornips E., Hesselink M.K., Schrauwen P. (2010). Exercise training increases mitochondrial content and ex vivo mitochondrial function similarly in patients with type 2 diabetes and in control individuals. Diabetologia.

[17] Grevendonk L., Connell N.J., McCrum C., Fealy C.E., Bilet L., Bruls Y.M.H. (2021). Impact of aging and exercise on skeletal muscle mitochondrial capacity, energy metabolism, and physical function. Nat Commun.

[18] Axelrod C.L., Fealy C.E., Mulya A., Kirwan J.P. (2019). Exercise training remodels human skeletal muscle mitochondrial fission and fusion machinery towards a pro-elongation phenotype. Acta Physiol.

[19] Fealy C.E., Mulya A., Lai N., Kirwan J.P. (2014). Exercise training decreases activation of the mitochondrial fission protein dynamin-related protein-1 in insulin-resistant human skeletal muscle. J Appl Physiol.

[20] Arribat Y., Broskey N.T., Greggio C., Boutant M., Conde Alonso S., Kulkarni S.S. (2019). Distinct patterns of skeletal muscle mitochondria fusion, fission and mitophagy upon duration of exercise training. Acta Physiol.

[21] Houzelle A., Jorgensen J.A., Schaart G., Daemen S., van Polanen N., Fealy C.E. (2021). Human skeletal muscle mitochondrial dynamics in relation to oxidative capacity and insulin sensitivity. Diabetologia.

[22] Dahl R., Larsen S., Dohlmann T.L., Qvortrup K., Helge J.W., Dela F. (2015). Three-dimensional reconstruction of the human skeletal muscle mitochondrial network as a tool to assess mitochondrial content and structural organization. Acta Physiol.

[23] Kristensen M.D., Petersen S.M., Moller K.E., Lund M.T., Hansen M., Hansen C.N. (2018). Obesity leads to impairments in the morphology and organization of human skeletal muscle lipid droplets and mitochondrial networks, which are resolved with gastric bypass surgery-induced improvements in insulin sensitivity. Acta Physiol.

[24] Axelrod C.L., Fealy C.E., Erickson M.L., Davuluri G., Fujioka H., Dantas W.S. (2021). Lipids activate skeletal muscle mitochondrial fission and quality control networks to induce insulin resistance in humans. Metabolism.

[25] Mishra P., Varuzhanyan G., Pham A.H., Chan D.C. (2015). Mitochondrial dynamics is a distinguishing feature of skeletal muscle fiber types and regulates organellar compartmentalization. Cell Metabol.

[26] Campos J.C., Marchesi Bozi L.H., Krum B., Grassmann Bechara L.R., Ferreira N.D., Arini G.S. (2023). Exercise preserves physical fitness during aging through AMPK and mitochondrial dynamics. Proc Natl Acad Sci U S A.

[27] Amaro-Gahete F.J., Jurado-Fasoli L., Trivino A.R., Sanchez-Delgado G., De-la O.A., Helge J.W. (2019). Diurnal variation of maximal fat-oxidation rate in trained male athletes. Int J Sports Physiol Perform.

[28] Robles-Gonzalez L., Aguilar-Navarro M., Lopez-Samanes A., Ruiz-Moreno C., Munoz A., Varillas-Delgado D. (2022). No diurnal variation is present in maximal fat oxidation during exercise in young healthy women: a cross-over study. Eur J Sport Sci.

