# Supplementary material for: Twenty-four hour rhythmicity in mitochondrial network connectivity and mitochondrial respiration; a study in human skeletal muscle biopsies of young lean and older individuals with obesity
Source: Mol Metab. 2023 Apr 14;72:101727. doi: 10.1016/j.molmet.2023.101727 (PMC10160594; doi:10.1016/j.molmet.2023.101727)
Supplement: Multimedia component 1 [file mmc1.pdf]

SUPPLEMENTAL INFORMATION

Supplemental Table 1. Participant characteristics

Parameter/group	Young healthy	Older metabolically impaired
Age (years)	22 ± 3	65 ± 9**
BMI (kg/m^2)	22.4 ± 2.0	30.3 ± 2.7**
Fasting plasma glucose (mmol/L)	NA	5.7 ± 0.4
Fasting plasma insulin (µIU/mL)	NA	13.8 ± 8.5
2-h plasma glucose (mmol/L)	NA	7.3 ± 1.5
HbA$_{1c}$ (%)	NA	5.3 ± 0.5
Glucose clearance (ml/kg/min)	NA	327 ± 38
MEQ-SA score	50 ± 7	57 ± 9*

*p<0.05; **p<0.01

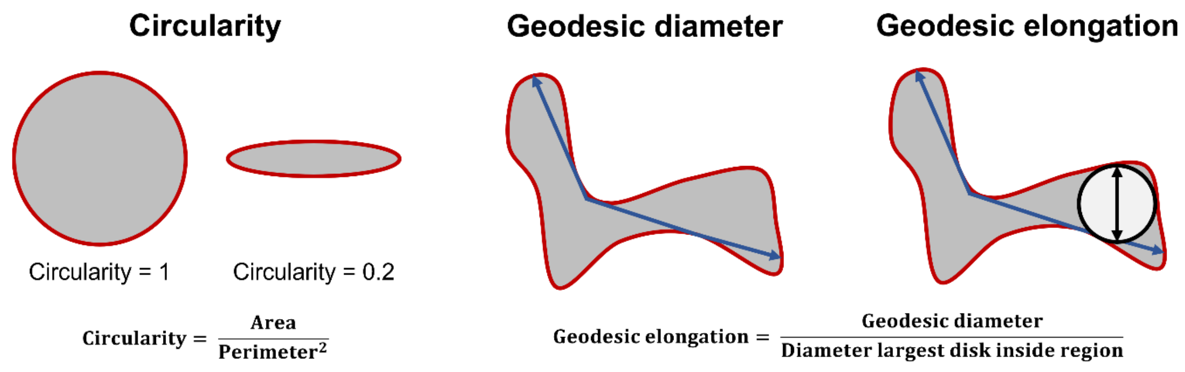

Supplementary Figure 1. Shape descriptors illustrating mitochondrial network morphology. Shape descriptors used to validate the MFI as a parameter describing the fragmentation status of the mitochondrial network. Punctate mitochondrial structures are characteristic for a fragmented mitochondrial network, while elongated structures are a feature of a fused mitochondrial network. Circularity describes whether a structure is a perfect circle or whether a structure is more elongated, ranging from 0 – 1; 0 describing an elongated structure and 1 describing a perfect circle. The geodesic diameter describes the larges geodesic distance between two points within a structure. The geodesic distance is the length of the shortest path joining two points while staying inside a structure. The geodesic diameter indicates the length of a structure. To draw conclusions whether a large geodesic diameter indicates an elongated structure the geodesic elongation needs to be calculated. The geodesic elongation is calculated by dividing the geodesic diameter by the diameter of the largest disk that fits inside the region. For a perfect circle the geodesic elongation is 1; the higher the geodesic elongation, the more elongated the structure. Red line = perimeter; blue arrow = geodesic diameter; black arrow = diameter largest disk fitting into region of interest.

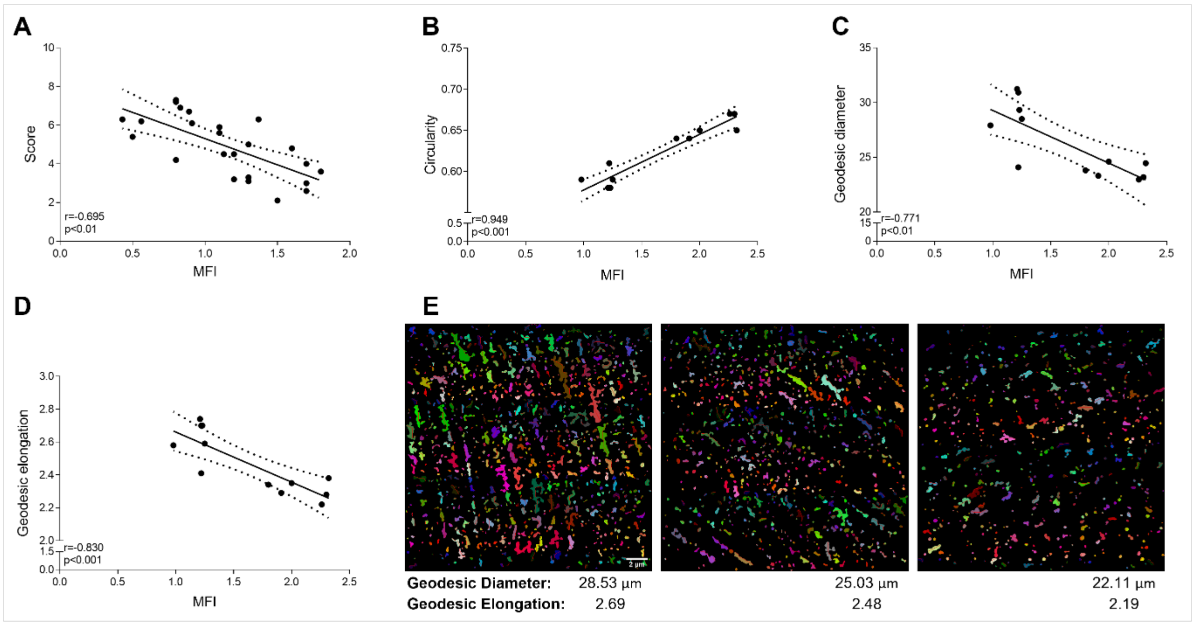

Supplementary Figure 2. Validation of the analysis tool. Correlation between MFI and visual scoring of the muscle mitochondrial network on a 10-point scale (average of two independent observers) (A), MFI and circularity (B), MFI and geodesic diameter (C), and MFI and geodesic elongation (D). (E) Object maps obtained from the analysis for the geodesic diameter and elongation. Images are shown with different levels of fragmentation and the corresponding average geodesic diameter and elongation for validation.

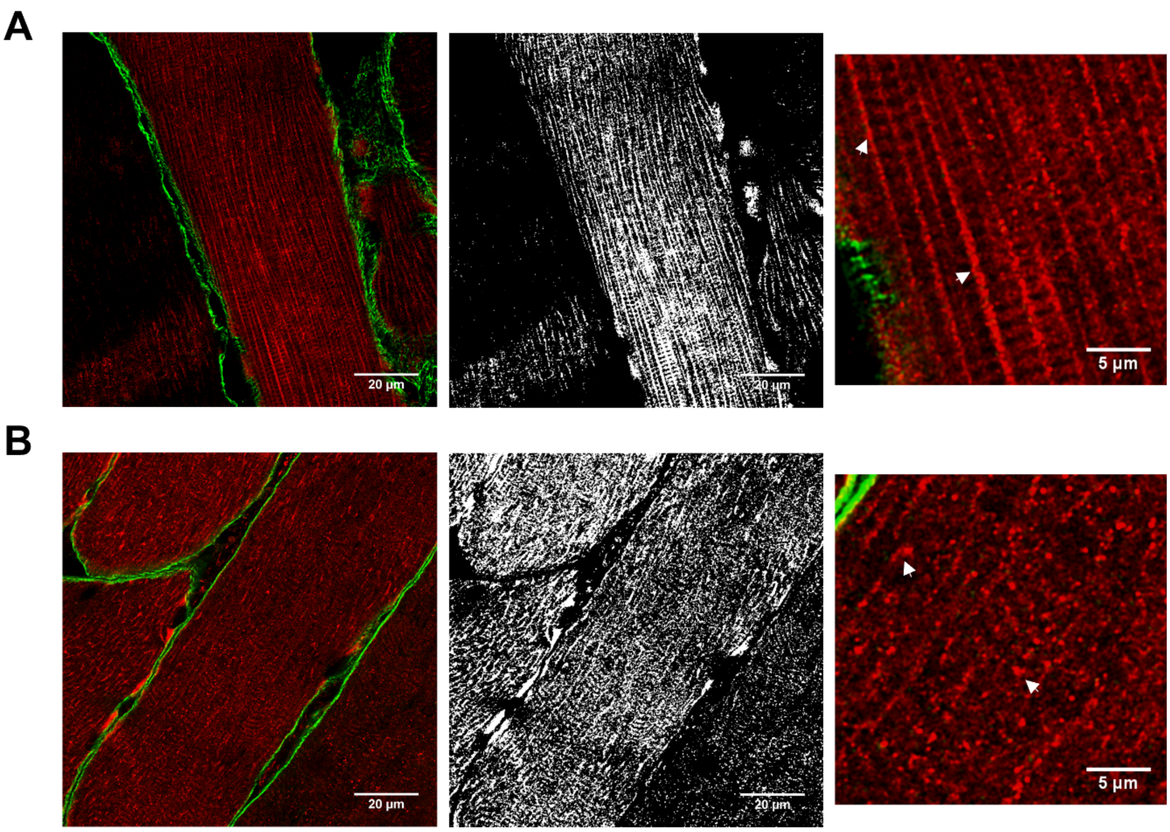

Supplementary Figure 3. Representative images used for validation of the analysis tool. Representative images and corresponding binary and zoomed images of muscle fibers with a high visual score (i.e. 9)/low MFI (A) and low score (i.e. 4.5)/high MFI (B) for the mitochondrial network. Arrows indicate either elongated mitochondrial structures (A) or punctate mitochondrial structures (B). Mitochondria are visualized in red and cellular membrane in green.
